# Evaluating tree biomass estimation in trans-Atlantic mangrove species: Comparing bole diameter measurements for improved accuracy

**DOI:** 10.1371/journal.pone.0323926

**Published:** 2025-09-11

**Authors:** Charles A. Price, Todd A. Schroeder, Benjamin Branoff, Humfredo Marcano-Vega, Nicole Pillot-Torres, Morgan Chaudry, Michael Ross, Monica Papeș, Skip Van Bloem

**Affiliations:** 1 Ecology and Evolutionary Biology, University of Tennessee, Knoxville, Tennessee, United States of America; 2 National Institute for Modeling Biological Systems, University of Tennessee, Knoxville, Tennessee, United States of America; 3 Forest Inventory and Analysis, Southern Research Station, USDA Forest Service, Knoxville, Tennessee, United States of America; 4 Boricorp, San Juan, Puerto Rico, United States of America; 5 Baruch Institute of Coastal Ecology and Forest Science, Clemson University, Georgetown, South Carolina, United States of America; 6 Institute of Environment, Florida International University, Miami, Florida, United States of America; Institute of Disaster Prevention, CHINA

## Abstract

Estimating the biomass of terrestrial forests generally, and mangrove forests in particular, is an area of considerable interest. Most approaches rely on empirically derived allometric models to predict tree biomass. The single parameter with the strongest predictive ability in most studies is diameter at breast height (DBH); however the use of DBH arose primarily out of convenience, not from an analysis of tree form. While DBH explains a lot of variability in other tree metrics such as height or above ground biomass, its utility in smaller species is uncertain. Here we used measurements from 302 destructively sampled mangrove trees of four species to test which of three bole diameter measurements, basal stem diameter (BSD), diameter at 30 cm (D30), and DBH, is the best predictor of aboveground biomass. D30 had the highest mean coefficient of determination (*R*^*2*^) and lowest mean root mean squared error (RMSE) across all site/species combinations. However, the improvement over DBH was modest, with a mean across all site/species combinations of 1.58 kg RMSE and *R*^*2*^ of 0.948 for D30, compared to 1.63 kg RMSE and *R*^*2*^ of 0.917 for DBH. Nevertheless, D30 may have utility in future studies as it allows for lower size thresholds and has better overall explanatory power than DBH.

## Introduction

Accurate prediction of the aboveground biomass of terrestrial trees underpins research at numerous levels, from interspecific resource exchange in tree communities to global models of forest dynamics [1]. Efforts to incentivize greenhouse gas emission reduction, sustainable forest management practices, and the adoption of blue carbon markets rely on accurate estimates of belowground and aboveground biomass [2,3]. Foresters and plant ecologists have long used diameter at breast height (DBH) to estimate biomass. However, the use of DBH as a standard measurement point emerged from a combination of convenience and sociocultural factors [4], rather than from an objective analysis of tree form. Moreover, the height at which DBH is measured differs across studies [5].

Despite these historical idiosyncrasies, DBH has been shown to correlate well with tree height and aboveground mass for large trees, particularly those with large boles growing in closed canopy forests [1]. However, for smaller woody trees and shrubs, it may not be the best predictor. Many small trees and shrubs branch extensively below DBH height, thus DBH for those plants captures the biomechanical and hydraulic investment for some, but not all of the plant. Foresters working in arid environments have long understood this and have measured the diameter at a woody plant's base instead of DBH as a correlate of above ground biomass. For example, the US Nationwide Forest Inventory (NFI) program [6] measures the diameter at root collar (DRC) for woodland species (e.g., pinyon-juniper, mesquite, oak woodlands, etc.) growing in arid regions of the western U.S. [7].

Mangrove species growing in the U.S. mainland and Caribbean islands exhibit a wide array of growth patterns, ranging from tall trees with large and easily measured boles to small shrubbier forms, sometimes referred to as dwarf mangroves [8]. Mangrove growth and tree size are known to vary at geographic scales [9,10], and can be influenced by surface hydrology and soil nutrient composition [11–13], temperature, humidity and salinity [14,15], and disturbance frequency and intensity [16,17]. Toward the northern extent of mangroves’ range, their growth is strongly influenced by cold temperatures, with shorter growth seasons leading to individuals that tend to be smaller and shrubbier [8,18].

As with other terrestrial forest systems, empirical allometric equations used to derive biomass have played a central role in the estimation of mangrove biomass from community to landscape scales [10] and are used to underpin carbon stock assessments [2,19]. Numerous studies worldwide have harvested mangrove trees and generated locally derived allometric functions [8,9,13,20–27]. While some studies have, out of necessity, included diameter measurement points lower than DBH [8,22], we are not aware of any attempts to systematically evaluate which of several possible measurement points has the greatest predictive power.

Here we analyze datasets from two sources that allows us to evaluate which of three measurement points at the basal end of trees is the best predictor of aboveground biomass. The first dataset (San Juan dataset) includes recent measurements for the linear dimensions (height and canopy diameter) and aboveground mass of 152 individual trees collected in San Juan, Puerto Rico [28]. The second dataset (Biscayne dataset) comes from an analysis of mangrove form and allometry using 150 individuals collected in Biscayne, Florida, USA [8]. Both studies contain data for the three true mangrove species growing regionally, *Avicennia germinans* (L.)L., *Laguncularia racemosa* (L.)C.F.Gaertn., and *Rhizophora mangle* L.; in addition, the San Juan dataset includes a common mangrove associate, *Conocarpus erectus* L. Tree diameter was measured at the base of the stem (BSD), at 30 cm above base height (D30), and at breast height (DBH) for both datasets, which we further analyze to determine the best predictor of aboveground mass.

## Materials and methods

The San Juan dataset was collected in November 2024 (see [28] for full methods). Collection methods for the Biscayne dataset can be found in Ross et al. [8]. As both studies collect similar measurements using similar methods, we only briefly summarize the protocol used to collect the San Juan dataset.

For each tree, diameter was measured at a point just above the butt swell (the thickening of the tree base at ground level to support the tree mass) (*BSD*), at 30 cm (*D30*) above the *BSD* measurements point, and at 137 cm above the *BSD* measurement point (referred to here as *DBH*). The height of each tree was measured with a tape measure (short trees) or a hypsometer (tall trees). The canopy diameter was measured at its widest point, and again at an axis perpendicular to the first canopy measurement. All trees were cut at ground level and the wet mass of each tree was measured with either a digital scale (small trees) or a spring scale (large trees) suspended from a tall aluminum step-ladder. For large trees, each individual was cut into several sections to enable weighing.

### Wood discs

Due to logistical constraints, we were unable to dry and weigh entire trees to determine dry biomass. Instead, we estimated the dry mass fraction from wood discs cut from branches of various sizes for each species; the discs were dried to constant mass and weighed to calculate the dry mass fraction as dry mass/wet mass. We then multiplied *wet mass* of the tree by the dry mass fraction of the wood discs to yield aboveground *dry mass* estimates. This approach is similar to that used by several other researchers reporting biomass allometries for these species [13,23–25,27].

### Allometric analyses

Bivariate relationships between all measurements (three bole diameter measures and dry mass) were estimated using ordinary least squares (OLS) regression. To estimate regression function variables and model fits we utilized the software package Standardized Major Axis Tests and Routines (SMATR) [29,30]. OLS regression is typically used in allometric studies where the goal is to predict the values of the Y-variable using the X-variable [30]. Data were log transformed prior to regression fitting to meet the assumption of homogeneity of variance. All regression functions were tested at the p < 0.05 significance level.

We fit OLS regression functions to multiple different groupings, both inter and intraspecifically, within and between sites. To examine patterns across both the Biscayne and San Juan data, we combined both datasets (referred to as Combined). We also analyzed intraspecific data both within (S1 Table) and across sites (Table 1). Not all trees had all three measurements as some trees were simply too small to measure DBH or even D30. To determine if our findings were influenced by samples with only one or two of the measurements (BSD or D30), we also generated regression functions using only trees with all three measurements (denoted with a “3” in Table 2). Raw data measures for each tree are in S2 Table. Regression results for the subset of trees that included all three measurements are in S3 Table.

**Table 1:** OLS allometric regression data. The first and second columns contain the Log10 transformed Y and X variables, respectively, followed by the dataset, group, sample size (n), R2 values, slope with lower and upper 95% confidence intervals, and intercept with lower and upper 95% confidence intervals. Results for the San Juan and Biscayne data “combined” are presented here. Results for each site individually are in S1 Table.

Log_10_ Y variable	Log_10_ X variable	Dataset	Group	n	R^2^	Slope	LowCI	UppCI	Interc	LowCI	UppCI
Dry Mass (kg)	BSD (cm)	Combined	none	298	0.893	3.190	3.064	3.316	−1.870	−1.946	−1.794
Dry Mass (kg)	D30 (cm)	Combined	none	239	0.951	2.430	2.359	2.501	−1.009	−1.052	−0.965
Dry Mass (kg)	DBH (cm)	Combined	none	153	0.907	1.804	1.711	1.897	−0.246	−0.308	−0.183
Dry Mass (kg)	BSD (cm)	Combined	*A. germinans*	65	0.969	3.167	3.025	3.308	−1.678	−1.760	−1.595
Dry Mass (kg)	BSD (cm)	Combined	*C. erectus*	32	0.954	2.376	2.181	2.571	−1.025	−1.162	−0.889
Dry Mass (kg)	BSD (cm)	Combined	*L. racemosa*	94	0.954	2.938	2.805	3.071	−1.932	−2.025	−1.840
Dry Mass (kg)	BSD (cm)	Combined	*R. mangle*	107	0.852	4.163	3.827	4.500	−2.319	−2.483	−2.155
Dry Mass (kg)	D30 (cm)	Combined	*A. germinans*	58	0.962	2.389	2.262	2.516	−0.980	−1.054	−0.906
Dry Mass (kg)	D30 (cm)	Combined	*C. erectus*	32	0.965	2.254	2.093	2.415	−0.796	−0.902	−0.691
Dry Mass (kg)	D30 (cm)	Combined	*L. racemosa*	59	0.976	2.270	2.175	2.365	−1.021	−1.096	−0.946
Dry Mass (kg)	D30 (cm)	Combined	*R. mangle*	90	0.941	2.769	2.623	2.916	−1.084	−1.155	−1.013
Dry Mass (kg)	DBH (cm)	Combined	*A. germinans*	39	0.949	2.007	1.851	2.162	−0.444	−0.538	−0.349
Dry Mass (kg)	DBH (cm)	Combined	*C. erectus*	27	0.884	1.708	1.453	1.963	−0.218	−0.381	−0.056
Dry Mass (kg)	DBH (cm)	Combined	*L. racemosa*	46	0.952	1.949	1.816	2.081	−0.451	−0.556	−0.346
Dry Mass (kg)	DBH (cm)	Combined	*R. mangle*	41	0.946	1.733	1.600	1.867	0.021	−0.060	0.103

**Table 2:** *RMSE* and *R*^*2*^ for different data groupings. The first column contains the species composition (inter, intra, or species specific) and the second column the data composition for each statistic reported in the remaining columns. Columns 3-5 contain the RMSE (kg), columns 6-8 the *R*^*2*^, and columns 9-11 the % error relative to the mean biomass in the dataset for BSD, D30, and DBH, respectively (see Methods). The subset of the data for trees that had all three measurements is denoted by the terms “Combined 3” and “Site 3” in the Data column. The lowest RMSE value in a row for each metric is in bold. Similarly, the highest *R*^2^ for each row is in bold. Values that represent means are underlined. The mean for each column and each grouping is given in the final two rows. The final row contains means for those trees with all three measures. The row above it contains means for all trees.

		RMSE (kg)			*R*^*2*^ (mean)			% Error relative to mean mass		
Group	Data	BSD	D30	DBH	BSD	D30	DBH	BSD	D30	DBH
Interspecific	Combined	2.946	**1.820**	1.828	0.893	**0.951**	0.907	20.520	**12.676**	12.736
Intraspecific	Combined	2.409	1.703	**1.616**	0.932	**0.961**	0.933	16.778	11.860	**11.259**
Interspecific	Site	2.523	**1.616**	1.763	0.880	**0.942**	0.764	17.573	**11.261**	12.280
Intraspecific	Site	2.082	**1.490**	1.503	0.926	**0.959**	0.937	14.505	**10.381**	10.472
*A. germinans*	Combined	1.737	1.649	**1.540**	**0.969**	0.962	0.949	12.100	11.486	**10.725**
*L. racemosa*	Combined	2.242	**1.576**	1.602	0.954	**0.976**	0.952	15.620	**10.977**	11.162
*R. mangle*	Combined	3.183	1.892	**1.582**	0.852	0.941	**0.946**	22.171	13.179	**11.019**
*C. erectus*	Combined	1.527	**1.448**	1.790	0.954	**0.965**	0.884	10.637	**10.089**	12.473
*A. germinans*	Site	1.721	1.466	**1.425**	0.967	**0.978**	0.963	11.991	10.211	**9.928**
*L. racemosa*	Site	1.820	**1.441**	1.503	**0.952**	0.948	0.936	12.679	**10.035**	10.470
*R. mangle*	Site	2.651	1.550	**1.352**	0.852	**0.947**	0.942	18.465	10.798	**9.418**
*C. erectus*	Site	1.527	**1.448**	1.790	0.954	**0.965**	0.884	10.637	**10.089**	12.473
Interspecific	Combined 3	2.808	**1.739**	1.821	0.856	**0.925**	0.909	19.563	**12.116**	12.686
Intraspecific	Combined 3	2.488	**1.573**	1.617	0.912	**0.957**	0.934	17.331	**10.960**	11.263
Interspecific	Site 3	2.371	**1.657**	1.756	0.736	**0.878**	0.771	16.517	**11.544**	12.234
Intraspecific	Site 3	2.009	**1.459**	1.506	0.847	0.934	**0.939**	13.994	**10.163**	10.491
*A. germinans*	Combined 3	1.991	**1.406**	1.614	0.952	**0.965**	0.949	13.870	**9.794**	11.246
*L. racemosa*	Combined 3	1.838	**1.536**	1.734	0.932	**0.971**	0.952	12.801	**10.703**	12.076
*R. mangle*	Combined 3	3.722	2.093	**1.863**	0.832	0.937	**0.949**	25.927	14.578	**12.982**
*C. erectus*	Combined 3	1.719	**1.456**	1.827	0.935	**0.956**	0.884	11.977	**10.146**	12.730
*A. germinans*	Site 3	1.583	**1.346**	1.425	0.935	**0.983**	0.963	11.028	**9.373**	9.928
*L. racemosa*	Site 3	1.482	**1.453**	1.511	**0.965**	0.960	0.935	10.324	**10.123**	10.528
*R. mangle*	Site 3	3.155	1.565	**1.349**	0.599	0.850	**0.947**	21.978	10.904	**9.398**
*C. erectus*	Site 3	1.547	**1.454**	1.790	0.935	**0.956**	0.884	10.778	**10.126**	12.473
Mean all trees		2.197	**1.592**	1.608	0.924	**0.958**	0.916	15.306	**11.087**	11.201
Mean trees with 3 measures		2.226	**1.561**	1.651	0.870	**0.939**	0.918	15.507	**10.878**	11.503

### Root mean squared error

To determine which measure of tree diameter (BSD, D30, DBH) was the best predictor of total aboveground biomass (dry mass), and to compare results between regression models at different grouping levels (interspecifically or intraspecifically) we calculated the root mean squared error (RMSE) using standard formula, which represents the mean difference between the values predicted by the regression model and the actual measured values.

### Diameter ratios

To examine whether there was a systematic change in the values of our three diameter measurements, relative to one another, we analyzed how their ratios (BSD/DBH, BSD/D30, D30/DBH) changed as a function of tree height. We also plotted the diameter measurements against one another to evaluate if regression slopes fit to those data (standardized major axis regression) differed from the simple null expectation that they have a slope of 1 (isometric change, i.e., shape is preserved with changes in scale).

## Results

We were able to collect 41 *R. mangle*, 40 *A. germinans*, 39 *L. racemosa*, and 32 *C. erectus* individual trees from the site in San Juan. The data from Ross et al. [8] contained measurements for 67 *R. mangle*, 26 *A. germinans*, and 57 *L. racemosa* individuals.

We collected wood sample discs from 27 *C. erectus*, 20 *A. germinans*, 14 *R. mangle,* and 10 *L. racemosa* individuals to generate dry mass fraction estimates. There were no significant differences between groups at the species level (one-way ANOVA, p = 0.417). As such, we used the mean dry mass fraction across all species (0.6046), as the basis for a correction factor of 60% of the *wet mass* for all trees as the estimate of their *dry mass*.

**Fig 1:**
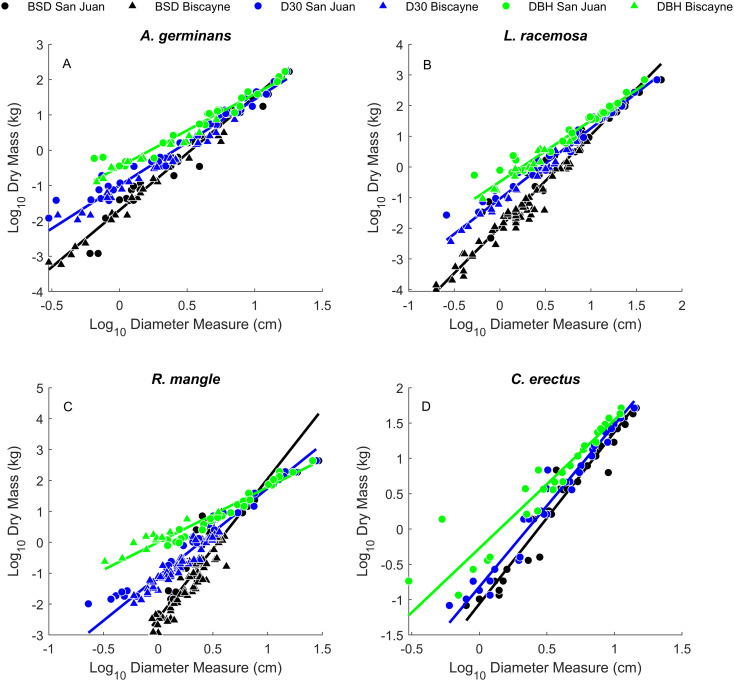
Bivariate relationships between the three diameter measurements (BSD, D30, and DBH) and above ground dry mass for the four mangrove species. Each panel corresponds to data for a given species as denoted in the panel title. Notice that the three functions appear to converge for larger trees in all four species.

**Fig 2:**
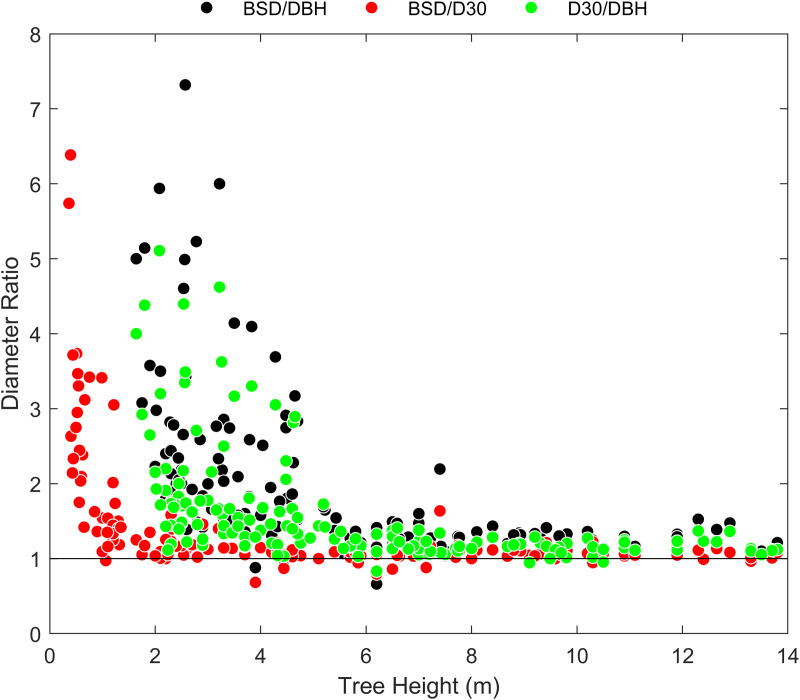
Diameter ratios as a function of tree height. As tree height increases, all three diameter ratios appear to approach a value just above 1, asymptotically. Note that both distributions involving DBH are constrained to begin above a minimum of 1.3 m (DBH height).

**Fig 3:**
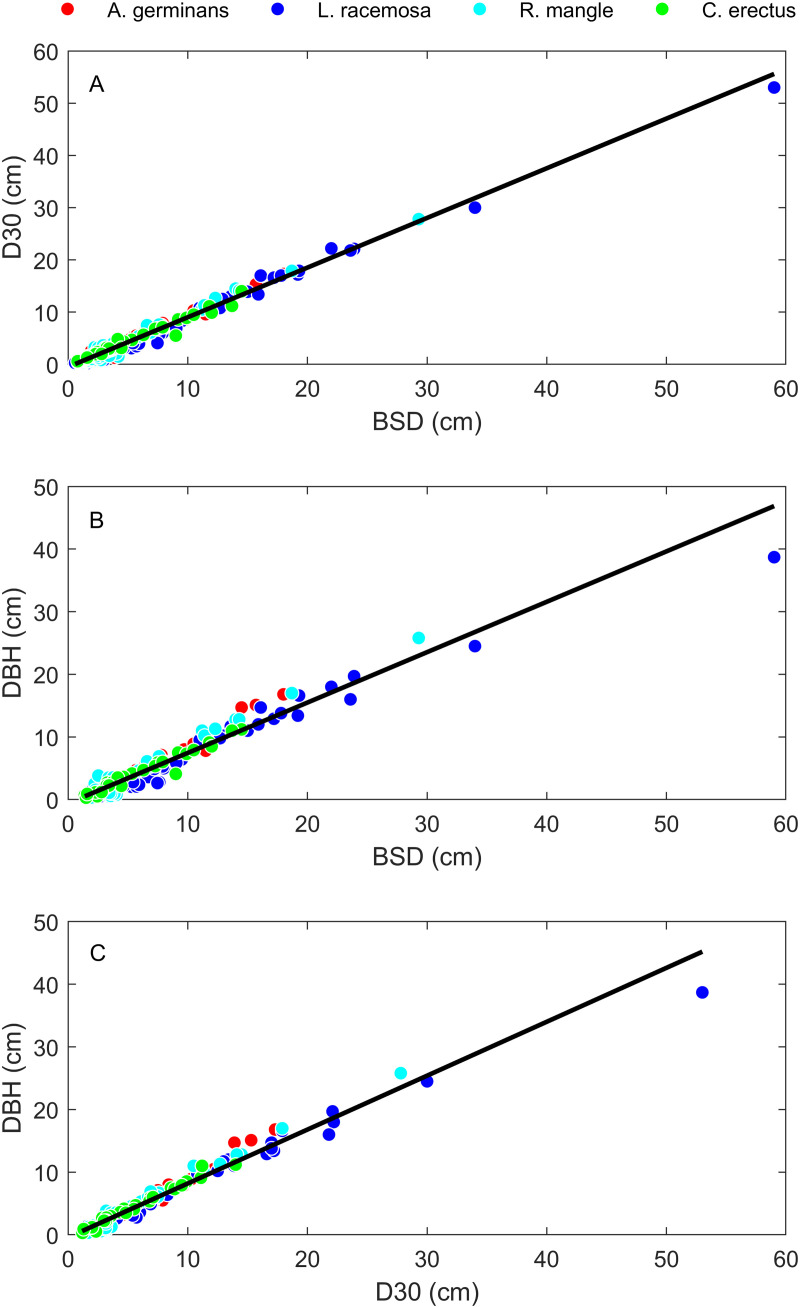
Allometric relationships between the three different diameter measurements. Pairwise bivariate relationships between D30 and BSD (Panel A), DBH and BSD (Panel B), and DBH and D30 (Panel C). Black lines represent interspecific SMA regression lines. Both interspecific and intraspecific regression data are given in Table 3.

Allometric regression results for the relationships between dry mass and our three different diameter measurements (BSD, D30, and DBH) are shown in Fig 1, Table 1, and S1 Table. Overall, there is very little variability around the fitted linear relationships between tree diameter and mass measurements. The mean *R*^*2*^ across all the relationships in Table 1 is 0.937. *R*^*2*^ values for different data groupings are provided in Table 2. Among the three diameter measurements, D30 had the highest *R*^*2*^ value in 17/24 cases and DBH had the highest *R*^*2*^ value in 7/24 cases. All three diameter ratios decreased with increasing tree size (Fig 2) and BSD, D30, and DBH were all strongly correlated with one another (Fig 3).

**Table 3:** SMA allometric regression data. Regression statistics for both interspecific and intraspecific bivariate relationships between BSD, D30, and DBH. Note that the slopes for *A. germinans* and *R. mangle* are not statistically different than isometry in all three cases, while those for *C. erectus* and *L. racemosa* are statistically different.

Y variable	X variable	Group	n	R2	Slope	LowCI	UppCI	Interc	LowCI	UppCI
Mean D30 (cm)	Mean BSD (cm)	Interspecific	238	0.986	0.951	0.936	0.965	−0.489	−0.608	−0.370
Mean DBH (cm)	Mean BSD (cm)	Interspecific	151	0.948	0.805	0.775	0.835	−0.600	−0.906	−0.295
Mean DBH (cm)	Mean D30 (cm)	Interspecific	153	0.971	0.861	0.837	0.885	−0.418	−0.640	−0.196
Mean D30 (cm)	Mean BSD (cm)	A. germinans	59	0.985	0.991	0.959	1.024	−0.475	−0.668	−0.282
Mean D30 (cm)	Mean BSD (cm)	C. erectus	32	0.97	0.904	0.847	0.964	−0.143	−0.535	0.249
Mean D30 (cm)	Mean BSD (cm)	L. racemosa	58	0.992	0.936	0.913	0.959	−0.589	−0.888	−0.291
Mean D30 (cm)	Mean BSD (cm)	R. mangle	89	0.978	1.027	0.995	1.060	−0.785	−0.968	−0.603
Mean DBH (cm)	Mean BSD (cm)	A. germinans	40	0.973	1.001	0.949	1.057	−1.440	−1.822	−1.059
Mean DBH (cm)	Mean BSD (cm)	C. erectus	27	0.957	0.818	0.751	0.891	−0.647	−1.156	−0.138
Mean DBH (cm)	Mean BSD (cm)	L. racemosa	44	0.965	0.751	0.709	0.796	−0.704	−1.353	−0.054
Mean DBH (cm)	Mean BSD (cm)	R. mangle	40	0.965	0.975	0.917	1.037	−1.173	−1.652	−0.695
Mean DBH (cm)	Mean D30 (cm)	A. germinans	40	0.982	1.030	0.985	1.076	−1.148	−1.450	−0.846
Mean DBH (cm)	Mean D30 (cm)	C. erectus	27	0.979	0.907	0.855	0.963	−0.538	−0.889	−0.188
Mean DBH (cm)	Mean D30 (cm)	L. racemosa	45	0.982	0.804	0.771	0.837	−0.261	−0.707	0.185
Mean DBH (cm)	Mean D30 (cm)	R. mangle	41	0.981	0.991	0.947	1.036	−0.963	−1.297	−0.629

Looking further at Table 2, D30 had the lowest RMSE in 17/24 cases, and DBH had the lowest RMSE in 7/24 cases,. As seen at the bottom of Table 2, the mean RMSE, *R*^2^, and relative error do not appear to be significantly affected by whether the regressions were performed on all measures, or just those trees with all three measurements.

As seen in Table 3, all interspecific slopes were less than isometry. However, half of the intraspecific slopes were not statistically different from the expectation for isometry, with slope 95% confidence intervals that include 1.

## Discussion

Knowledge of the biomass contained in trees at individual, community, landscape, and global levels has been a central focus of ecological research for decades. Model-derived estimates of above ground biomass inform our understanding of community and ecosystem dynamics and guide management decisions. Empirically derived allometric relationships have been a cornerstone of such modeling approaches for over a century [1,31].

The use of DBH as a biomass predictor has been central to such approaches and is frequently the most informative variable used in predicting tree biomass, when compared to other tree measurements such as tree height or canopy spread [1]. However, there are limitations in using DBH for smaller, shrubbier trees. Foremost among these is the fact that trees in many forests are simply too small to have DBH measured and are typically ignored in censuses or subsampled in small areas. This is despite the fact that small trees can contribute substantially to biomass estimates, particularly in disturbed areas or forest types containing smaller tree species such as mangroves. Further, many trees branch extensively below the DBH measurement point, thus DBH for those trees captures only some of the hydraulic and biomechanical investment required for distal tree branches and is a weaker correlate of total aboveground biomass.

To that end, we compared the predictive power of three different measurements of the diameter of mangrove trees: BSD, D30, and DBH. Looking at the amount of variance explained by the different regression functions (*R*^*2*^), those based on D30 explain more variability than BSD or DBH in ~71% of cases (Table 2). Moreover, the *R*^*2*^ for both interspecific regressions (within each site and across sites), and the mean *R*^*2*^ for intraspecific regressions are higher for D30 (Table 1). *R*^*2*^ values alone would suggest that D30 is a more adequate variable to measure for biomass estimation.

However, the results using RMSE are somewhat more equivocal. D30 does have the lowest RMSE in ~71% of the cases, compared to ~29% for DBH (Table 2). The mean percentage error relative to the mean mass for the three variables is lower for D30 (10.98%) compared to that for DBH (11.35%) and BSD (15.41%; Table 2), but only marginally.

Collectively, these results suggest that D30 has a better overall performance than DBH, but not by a lot, and in some cases, at the intraspecific level, DBH has lower RMSE and higher *R*^*2*^ values. We used *R*^*2*^ and RMSE as complimentary statistics. *R*^*2*^ is informative about the proportion of the variance explained by the regression model whereas RMSE represents the average magnitude of error between the actual and predicted values. Although the two metrics tend to be correlated, it is possible to have a regression model with a high *R*^*2*^ value that also has large RMSE values. This is because *R*^*2*^ is a relative measure of the proportion of total variance, while RMSE is a measure of the absolute difference between the model and the data.

As seen in Fig 1, the regression functions for all four species are divergent at small tree sizes, but start to converge as trees become larger, with smaller relative differences between diameter measurements (BSD, D30, DBH). This is also reflected in Fig 2: the diameter ratio values trend larger at small tree sizes, but appear to converge asymptotically to values just above 1 in larger trees. This indicates that the magnitude of the differences in these measurements decreases as trees become large.

All interspecific SMA regression lines had slopes <1 (Table 3). However, for two of the four species (*A. germinans* and *R. mangle*), all three bivariate relationships had intraspecific regression slopes that did not differ from isometry. This suggests the species may differ in shape changes during tree growth. Subsequent inquiries utilizing more comprehensive shape analyses such as with photographs of tree trunks, or terrestrial laser scanning, may provide more insight into the extent to which different mangrove species change shape systematically as they grow, and how this might influence efforts to find reliable proxies for whole plant biomass.

Allometric relationships are known to be variable, particularly when sample sizes are low [32,33], multiple variables are used to predict mass [34], or when examining allometric trends across resource gradients [10,35]. Further, the statistical model one chooses to characterize the relationship can also influence results. Several authors have examined how assumptions about the error structure and whether or not it is primarily in the Y-variable, or both the X and Y-variables, affect whether to choose OLS or SMA methods [30,36]. Moreover, in situations where prior information regarding parameter distributions can inform error structure modeling, or where non-linear or complex hierarchical data structures make inference challenging, Bayesian approaches have been found to be useful [37,38].

Our combined dataset includes 302 trees across four species, which is reasonably large for allometric studies in mangroves, but could benefit from increased samples from more species and a range of sites to help validate our findings. In addition, our simplifying assumption that dry biomass is 60% of fresh biomass, while supported by methods similar to those many groups have used [13,23–25,27], could also benefit from greater sampling. Branch water content is known to be influenced by branch size [39], and while we sampled a range of diameters, our sample sizes were not large enough to allow for a closer look at this issue. Moreover, the distribution of branch sizes within each individual tree is unknown. Future efforts to examine whole tree water content, or the distribution of branch sizes and their water storage content within and across mangrove trees of differing size, would help to understand how tree and branch size influence water content.

In light of our results we recommend that future studies collecting allometric data for these and other mangrove species consider incorporating multiple measurements of bole diameter so that more data can inform the issue. Measuring D30 takes the same amount of time as DBH, thus it is unlikely to add substantial time or effort to field data collection. Since most of the trees in our data compilation are toward the smaller end of the size spectrum, it would be extremely informative if future studies collected these measurements from areas where these species attain their maximum size. At the very least, censuses using D30 can lead to larger sample sizes by allowing a lower size threshold, particularly in young or regenerating stands. Adding more small trees will help better resolve their contribution to total mass, thus potentially leading to more informed size thresholds for future sampling efforts.

## Supporting information

S1 Table OLS allometric regression results for the San Juan and Biscayne datasets, individually. The first and second columns contain the Log10 transformed Y and X variables, respectively, followed by the dataset, group, sample size (*n*), *R*^2^ values, slope with lower and upper 95% confidence intervals, and intercept with lower and upper 95% confidence intervals. Thick borders are included to help the reader identify data analyzed at the same level.
(XLSX)

S2 Table Raw tree measurements and predicted mass. Columns in order contain the dataset, species, mean BSD, D30 and DBH measures, tree height, above ground dry mass (kg), and mean canopy diameter (m). Columns J-U contain the predicted mass based on allometric equations given in Table 1, using interspecific combined data (columns J-L), intraspecific combined data (columns M-O), interspecific site data (columns P-R), and intraspecific site data (Columns S-U).
(XLSX)

S3 Table OLS regression results for a subset of trees that had all three measurements (BSD, D30 and DBH). The first and second columns contain the Log10 transformed Y and X variables, respectively, followed by the dataset, group, sample size (n), R2 values, slope with lower and upper 95% confidence intervals, and intercept with lower and upper 95% confidence intervals. Thick borders are included to help the reader identify data analyzed at the same level.
(XLSX)
